# Intercalation of CO_2_ Selected by Type of Interlayer Cation in Dried Synthetic Hectorite

**DOI:** 10.1021/acs.langmuir.2c03093

**Published:** 2023-03-29

**Authors:** Kristoffer W. Bø Hunvik, Konstanse Kvalem Seljelid, Dirk Wallacher, Alexsandro Kirch, Leide P. Cavalcanti, Patrick Loch, Paul Monceyron Røren, Paulo Henrique Michels-Brito, Roosevelt Droppa-Jr, Kenneth Dahl Knudsen, Caetano Rodrigues Miranda, Josef Breu, Jon Otto Fossum

**Affiliations:** † Department of Physics, Norwegian University of Science and Technology, N-7491 Trondheim, Norway; ‡ Helmholtz-Zentrum-Berlin, 14109 Berlin, Germany; § Departamento de Física dos Materiais e Mecânica, Instituto de Física, Universidade de São Paulo, 05508-090 São Paulo, SP Brazil; ∥ Institute for Energy Technology (IFE), 2007 Kjeller, Norway; ⊥ Bavarian Polymer Institute and Department of Chemistry, University of Bayreuth, 95447 Bayreuth, Germany; # Universidade Federal do ABC (UFABC), Av. dos Estados, 5001 - Santa Terezinha, Santo André, SP CEP 09210-580, Brazil

## Abstract

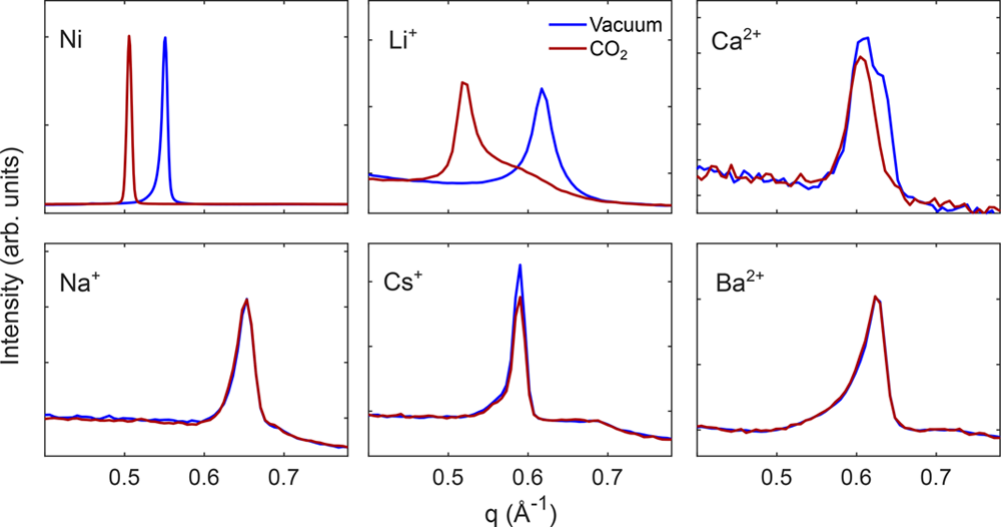

Clay minerals are abundant in caprock formations for anthropogenic storage sites for CO_2_, and they are potential capture materials for CO_2_ postcombustion sequestration. We investigate the response to CO_2_ exposure of dried fluorohectorite clay intercalated with Li^+^, Na^+^, Cs^+^, Ca^2+^, and Ba^2+^. By *in situ* powder X-ray diffraction, we demonstrate that fluorohectorite with Na^+^, Cs^+^, Ca^2+^, or Ba^2+^ does not swell in response to CO_2_ and that Li-fluorohectorite does swell. A linear uptake response is observed for Li-fluorohectorite by gravimetric adsorption, and we relate the adsorption to tightly bound residual water, which exposes adsorption sites within the interlayer. The experimental results are supported by DFT calculations.

## Introduction

Understanding how carbon dioxide may adsorb in the interlayers of clay minerals and tuning these mechanisms could result in pathways for mitigating greenhouse gas emissions. Smectite clays both adsorb CO_2_^1−14^ and are present in caprock formations for the storage of anthropogenic CO_2_;^15^ however, the full mechanisms for CO_2_ adsorption in these materials are still unresolved.

Smectites are nanolayered phyllosilicates that consist of two tetrahedral sheets sandwiching an octahedral sheet, where isomorphic substitutions in these sheets lead to a negative layer charge that is compensated for by interlayer cations. These interlayer cations are readily hydrated, depending on the type of cation, and swelling in response to humidity occurs.^16−18^ In addition, CO_2_ may intercalate and result in crystalline swelling.^3,5,8,11,19,20^

The swelling response to CO_2_ for smectites has received considerable attention in the literature. The swelling of hydrated clays and swelling in response to wet-supercritical CO_2_ have shown that a small amount of humidity enhances the CO_2_ adsorption; however, at high relative humidities, the CO_2_ is displaced by water, as the solvation energy is higher for water than for CO_2_.^21−28^

When the clay is dehydrated, open questions remain. For Wyoming montmorillonite (SWy-2) exchanged with Na^+^, NH_4_^+^ and Cs^+^ swelling is observed only for the larger cations under dry supercritical CO_2_ exposure.^11^ In ref (29), where hectorite with a mix of F^–^ and OH^–^ substitutions is studied, swelling is observed with Cs^+^ as the interlayer cation, not for the smaller cation Na^+^. From simulations on OH-hectorite,^29^ it is argued that smaller cations such as Li^+^, Na^+^, Mg^2+^, and Ca^2+^ have a global energy minimum in the collapsed zero water layer state (0WL). For larger cations such as K^+^, Rb^+^, Cs^+^, Sr^2+^, and Ba^2+^, a global energy minimum is found for the monolayer state (1WL), and the clay with these cations will spontaneously intercalate CO_2_. On the other hand, for dried synthetic fluorohectorite (Hec) it has been reported that with Li^+^ and Na^+^ as the interlayer cations the clay swells in response to CO_2_;^3,4^ however, for Na-Hec it was later shown that this is not always the case.^30^ It is argued that the mechanism behind this is that the interlayer is “propped open” by the cation size and thus the interlayer spacing is sufficiently large for adsorption.^11,20^ Cations with a size comparable to a CO_2_ monolayer, in particular, Cs^+^ and tetramethylammonium (TMA^+^), have been shown for anhydrous montmorillonite (SWy-2) to provide an optimum cation size for the highest CO_2_ adsorption.^20^ Cations larger than this optimum size either reduce the number of available adsorption sites by their larger occupancy or smaller cations compromise the accessibility due to their smaller size and require low temperatures and/or high pressures for CO_2_ adsorption.^20^ Water intercalation is controlled by the hydration energy of the cations;^18,31−33^ however, for CO_2_ intercalation, it has been argued that the interaction between the cation and the clay T–O–T (tetrahedral–octahedral–tetrahedral) structure plays a more important role.^11,29^

Hec is a synthetic and naturally occurring clay mineral that can be synthesized in a melt, resulting in large defect-free particles with a high charge homogeneity,^34^ providing an excellent template for studying interactions with CO_2_ without the interference of defects or impurities occurring in natural Hec. In previous works, we have shown that dried synthetic Ni-Hec readily adsorbs CO_2_^5^ and that this can be controlled by the layer charge magnitude,^19^ where a lower layer charge results in a higher adsorption capacity and a lower pressure threshold for adsorption.^19^ This CO_2_ sorption capacity has been related to an ordered corrensite-like structure with alternating layers of hydrated Ni^2+^ cations and a condensed nickel hydroxide phase.^5,35^ Here we study a synthetic Hec with only fluorine groups, whereas natural Hec has varying compositions of F^–^ and OH^–^ groups. Previous simulations^10^ have shown that for increasing F^–^ over OH^–^ substitutions, the CO_2_/(CO_2_ + H_2_O) ratio increases and the total amount of CO_2_ + H_2_O decreases.

Complete dehydration of smectite clays is challenging without venturing into a high-temperature region where the Hofmann–Klemen effect may occur.^36,37^ Therefore, more systematic studies are required to investigate dehydrated clay minerals with different interlayer cations in response to CO_2_. In the present work, we combine powder X-ray diffraction (PXRD) and gravimetric adsorption to study the swelling and uptake of CO_2_ by dried Hec exchanged with the interlayer cations Li^+^, Na^+^, Cs^+^, Ca^2+^, and Ba^2+^ in order to reveal the driving mechanisms for swelling in response to CO_2_. The experimental interpretations are fully supported by DFT calculations.

## Experimental Section

### Sample Preparation

Na-Hec with the stoichiometric composition of Na(Mg_5_Li)Si_8_O_20_F_4_ was prepared via melt synthesis according to a published procedure,^38^ followed by annealing (6 weeks, 1045 °C) to improve charge homogeneity and phase purity.^34^ To ensure the complete exchange for the different cations, Na-Hec was cation exchanged according to a published procedure^39^ using an *n*-hexylamine solution, followed by treatment with a 2 M solution of LiOH, CsOH, Ca(OH)_2_, and Ba(OH)_2_. Ni-Hec was prepared by exchange with nickel acetate according to a published procedure.^35^

### Thermogravimetric Analysis

The samples were characterized at NTNU with a Netzsch STA F3 449 Jupiter TGA measuring from 25 to 900 °C with a heating rate of 10 K/s under N_2_ flow (20 mL/min purge, 10 mL/min protective)

### X-ray Diffraction

X-ray diffraction patterns were collected at the XRD2 beamline at the Brazilian Synchrotron Light Laboratory, LNLS. A fixed wavelength of 1.305 Å was used, and the beam size was 0.3 mm × 0.3 mm. The scattering intensity was recorded by a Pilatus 300K area detector placed 114.8 mm from the sample. The 2D diffractograms were integrated using the software DAWN Science.^40^ To control the temperature, an Oxford Cryojet5 was fixed at the sample position. The samples were injected into a high-pressure capillary cell, which was customized based on the design in ref (41). The cell was connected to a gas handling system with vacuum controlled by a HiCube turbomolecular pump and gas pressure from a bottle of CO_2_ with a purity of 99.995%. The samples were heated in the capillaries to 150 °C under vacuum for drying, and the final drying was evaluated on the basis of the positions of the Bragg reflections. Samples were cooled to −20 °C, and if no peak movement was detected, then a pressure of 50 bar of CO_2_ was applied while recording continuously.

Additional X-ray diffraction measurements were carried out at NTNU (Trondheim, Norway) using an in-house X-ray scattering instrument attached to a Xenocs stationary electron impact source with a copper anode, producing Kα radiation. The scattering intensity was recorded by a two-dimensional Dectris Pilatus3 R 200K detector. The samples were mounted in the same sample cell in contact with a temperature-regulated copper plate.^42^ Temperature control was provided by Peltier elements, heat cartridges, and a circulation bath. The sample cell was connected to a gas handling system (Teledyne ISCO 260D) providing vacuum from a rotary vane pump (10^–2^ mbar) and pressurized CO_2_ of 99.9992% purity.

### Gravimetric Adsorption

CO_2_ adsorption measurements were conducted with an IsoSORP gravimetric sorption analyzer from Rubotherm. Each sample was prepared by degassing at 120 ± 5 °C overnight under high vacuum and was considered to be dry when no more mass loss occurred. For each sample, two measurements were conducted with an equilibration time of 1 or 4 h at each pressure step from high vacuum to 35 bar. The temperature was measured with a Pt-100 temperature sensor placed directly underneath the sample crucible surrounded by a double-walled thermostat controlled by a CC-K6 circulating water bath from Huber. The temperature stability of the sample was within 22.5 ± 1 °C. The suspension balance has a resolution of 0.01 mg and a reproducibility of <0.002% rdg (∼0.002 mg). The data for pressure, temperature, and sample weight were continuously recorded. The measured quantity is the excess adsorbed amount, which is obtained by correcting for the buoyancy of the skeletal volume of the sample material and the suspended metal parts (including the sample holder). The skeletal volume of the samples was determined by individual helium isotherms. The buoyancy of the suspended metal parts was obtained by a blank measurement with liquid CO_2_. The density of helium and CO_2_ for the given pressure and temperature conditions was obtained from the equation-of-state data provided by NIST.^43^

### DFT Calculations: Methodology

Following the same methodological protocol described in our recent studies,^5,19,30^ we perform first-principles calculations within the framework of density functional theory (DFT)^44,45^ as implemented in the SIESTA package.^46^ We considered a mesh cutoff of 400 Ry and a double-ζ plus polarization (DZP) basis set to represent the atomic charge in real space. The calculations were performed using the long-range van der Waals interactions implemented through the Berland and Hyldgaard^47^ exchange-correlation functional. Also, we included the slab-dipole correction to minimize the spurious dipole effects emerging from the charge rearrangement observed in the system. Atomic positions of the interlayer species and molecules were established by minimizing the forces and energies until the residual forces were less than 0.01 eV/Å. The cohesive energy between clay layers is a measure of the energy needed to separate the layers from each other. To determine this energy within the DFT approach, we consider the interaction energy between the layers as a function of their separation distance, *z*. This interaction energy curve is calculated by comparing the total energy of the system at a given separation distance *z* perpendicular to the material’s surface to a reference energy value obtained at a 100 Å separation layer distance (where the interaction between the layers is assumed to be minimal and may be neglected). This allows us to determine the energy required to separate the clay layers from each other, with those being at a given separation distance of *z*, and therefore compare the effect of each selected interlayer atom species on the cohesion. In addition to the cohesion energy, we also evaluated the adsorption energy of the CO_2_ and H_2_O molecules placed on top of the clay surface. To isolate the clay–molecule interactions from contributions in the adsorption energy coming from the spacial confinement, we consider a molecular model for the clay layer created by adding a vacuum in the *z* direction perpendicular to the exposed surface. The vacuum given is sufficiently large, so the interaction between repeated surfaces toward the *z* axis decreases to the state where it may be ignored. Then, the molecular adsorption energy was evaluated by *E*_Ads_ = *E*_total_ – (*E*_clay_ + *E*_molecule_), where *E*_total_ is the interacting system total energy and *E*_clay_ and *E*_molecule_ are the total energies of the isolated clay with interlayer ions and gas molecules, respectively. The adsorption energy calculated in such a way shows the affinity between the clay surface and the foreign molecules (H_2_O and CO_2_), which may help to interpret, together with the cohesion energy, the role of interlayer atomic species in the interlayer physicochemical environment.

## Results and Discussion

### X-ray Diffraction

**Figure 1:**
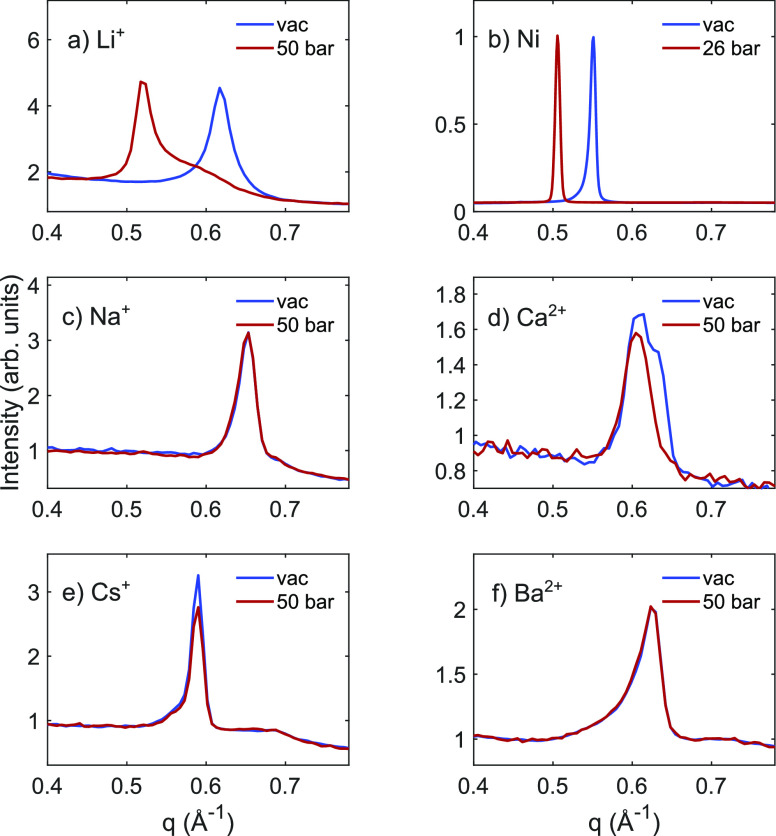
XRD pattern of the 001 peak in its dried state under vacuum and in response to 50 bar (26 bar for Ni-Hec) of CO_2_ at −20 °C for the respective cations in Hec with (a) Li^+^, (b) Ni, (c) Na^+^, (d) Ca^2+^, (e) Cs^+^, and (f) Ba^2+^. Ni-Hec was measured at SNBL BM01 at ESRF with the procedure given in ref (5). The remaining samples were measured at LNLS.

The samples were cation exchanged by six different interlayer cations with different sizes and valencies and therefore different potentials to polarize the CO_2_ molecules. In Figure 1, the dried state (heated to 150 °C for at least 1 h) and the final state at 50 bar of CO_2_ at −20 °C (liquid CO_2_) are shown for Hec with different cations. Their respective basal spacings between silicate layers are summarized in Table 1.

**Table 1:** Basal Spacing of Hec with the Indicated Cations in the Dried and CO_2_ Exposed States

cation	dried (Å)	CO_2_ exposed (Å)
Li^+^	10.2	12.2
Na^+^	9.6	9.6
Cs^+^	10.7	10.7
Ca^2+^	10.0 and 10.3	10.3
Ba^2+^	10.1	10.1

For the present work, Ni-Hec can be considered to be a reference, where the sample starts with a d_002_ of 11.4 Å in its dried state and swells to a final state of 12.3 Å. This swelling has previously been demonstrated in refs (5) and (19) to be due to the interaction with an ordered interstratified nickel hydroxide species.

The most evident result is the swelling for Li-Hec (Figure 1a), where it is initially observed at a basal spacing of 10.2 Å and swells to a final state of 12.2 Å. This is in full agreement with previous observations,^4^ demonstrating that CO_2_ is intercalated into Li-Hec. The asymmetric structure of the final Bragg reflection with a significant weight toward higher *q* values indicates that CO_2_ is not fully intercalated in all interlayers under these experimental conditions.

Na-Hec (Figure 1c), which is observed to have a basal spacing of 9.6 Å, in line with previous observations of dehydrated Na-Hec,^17^ does not swell in response to CO_2_, and there are no changes in the peak observed after CO_2_ exposure, again confirming our previous reports.^30^

Upon drying of Ca-Hec, the Bragg reflection forms a double-peak structure with seemingly two phases (Figure 1d) at 10 and 10.3 Å, with a combined peak width significantly broader than that of the other ions investigated. After exposure to CO_2_, the Bragg reflection forms a single symmetric peak at 10.3 Å. This suggests that Ca-Hec was not fully dehydrated and some residual water was left in the sample, producing two different environments with complete and incomplete dehydration. After CO_2_ exposure, this was homogenized possibly through a pressure effect or by the diffusion of water in the CO_2_ fluid phase. The absence of swelling from the initial state suggests that CO_2_ is not intercalated to any significant extent in this case. Cs-Hec is initially found at a basal spacing of 10.7 Å (Figure 1e), in line with previous observations of a basal spacing of 10.8 Å for dried Cs-Hec.^48^ This case also shows no significant response to CO_2_. Finally, for Ba-Hec at an initial basal spacing of 10.1 Å (Figure 1f), no changes in the response to CO_2_ are observed. This suggests that CO_2_ is not intercalated into the clays with Na^+^, Cs^+^, Ca^2+^, and Ba^2+^, as intercalation chemistry dictates that the swelling must accommodate the real size of the CO_2_ molecule (3.3 Å),^49^ which neither of these do (e.g., for Cs-Hec 10.7–9.6 Å = 1 Å < 3.3 Å).

**Figure 2:**
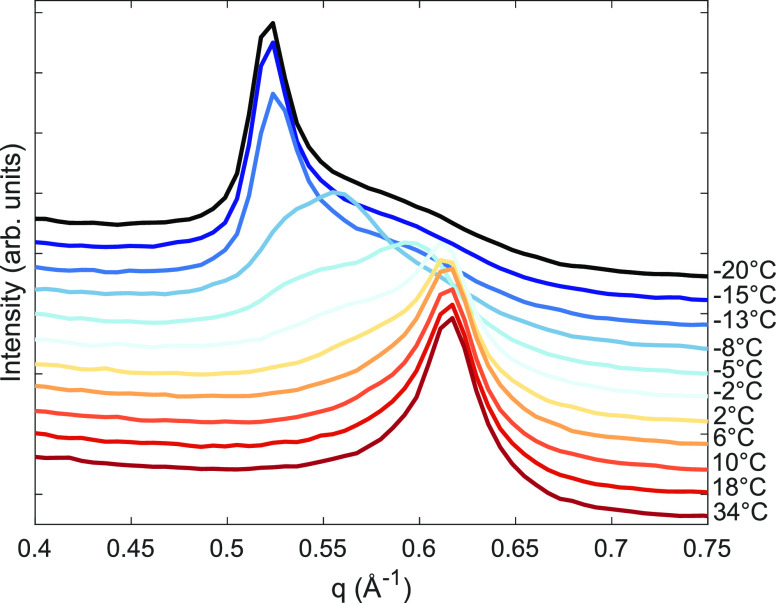
Powder X-ray diffraction of Li-Hec after reducing the pressure from 50 to 1 bar of CO_2_ and subsequently heating the sample as indicated. The curves have been shifted vertically by an offset for clarity. Experiments were carried out at LNLS.

Li-Hec shows a response to CO_2_ and remains in the swollen state even after reducing the CO_2_ pressure to 1 bar (Figure 2), which is similar to what has previously been observed for this material.^4^ Upon heating the sample, the basal spacing slowly shrinks with time and temperature, and after returning to ambient temperature, the Bragg reflection has returned to its initial dried position. This indicates some retention of CO_2_ at temperatures below 0 °C and that CO_2_ is completely released from the sample at approximately room temperature.

**Figure 3:**
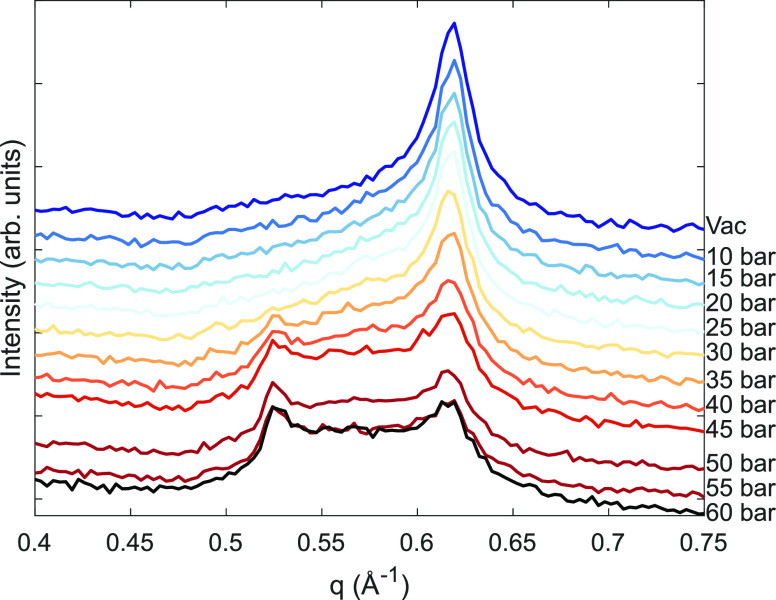
Powder X-ray diffraction of Li-Hec dried at 150 °C for 2.5 h and measured as a function of pressure for 1 h at each pressure step at 23 °C. The curves have been shifted vertically by an offset for clarity. Experiments were carried out at NTNU.

In the measurements described above, a full saturation of the sample in the CO_2_ liquid phase is studied. When exposed to CO_2_ gas at 23 °C, the evolution of the (001) Bragg reflection is as observed in Figure 3. There is slow growth as a function of the pressure of a peak at lower *q* values, which is the final product phase growing at the expense of the educt phase. Unlike Ni-Hec, which has previously been observed to have a clear pressure threshold before the full swollen state,^5^ Li-Hec swells continuously in a nearly linear fashion over this pressure range. In accordance with the measurements at −20 °C (Figure 1a) at the final pressure, there is still intensity left in the educt phase, indicating a partial incorporation of CO_2_ in the material. The gradual shift indicates that there is a random interstratification of CO_2_ within the interlayers. The observations may suggest that the measurements are not at equilibrium or that there are sites in the interlayers where adsorption is hindered.

### Gravimetric Adsorption Measurements

**Figure 4:**
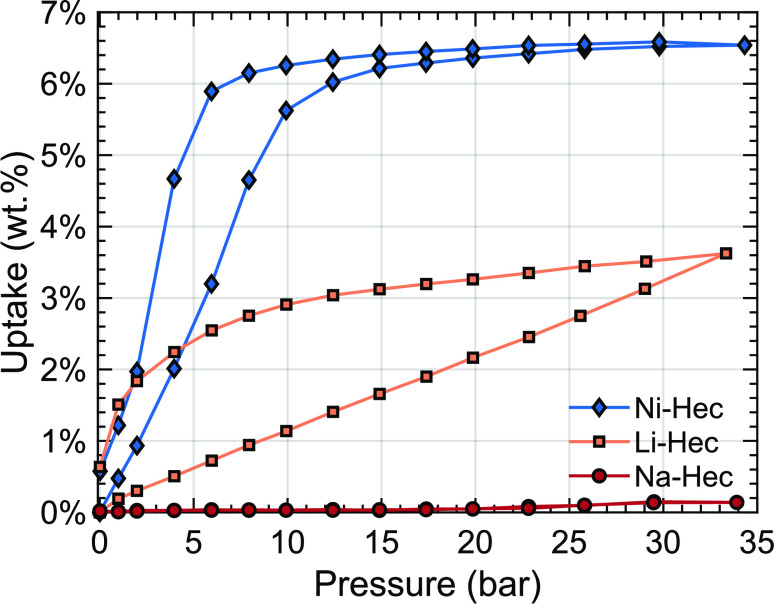
Gravimetric adsorption measurements of Na-Hec, Li-Hec, and Ni-Hec equilibrated at each pressure step for 4 h.

The gravimetric adsorption measurements were carried out only for Ni-Hec, Na-Hec, and Li-Hec and are shown in Figure 4. He isotherms were obtained over the same pressure range, giving densities of 2.35, 2.49, and 2.59 g/mL for Ni-Hec, Li-Hec, and Na-Hec, respectively. This is somewhat lower than the theoretical density of 2.8 g/mL for Na-Hec^17^ and suggests that parts of the sample are inaccessible to He.

Similar to the PXRD measurements, there is no response in the gravimetric adsorption measurements for Na-Hec. The small hump around 30 bar for the Na-Hec measurement is within the error of the measurement. For Li-Hec, on the other hand, a linear response as a function of pressure is observed for the uptake, followed by hysteresis behavior upon reducing the CO_2_ pressure. Ni-Hec shows a response in accordance with slitlike pores opening up and accepting CO_2_ homogeneously.^19^ Li-Hec, on the other hand, follows a linear C-type adsorption isotherm.^50^ Given the observations from XRD under the same conditions (Figure 3), this suggests that the swelling is not complete at 33 bar and that the increasing trend in uptake will hold at higher pressures. Upon desorption, the sample releases the CO_2_, demonstrating that interaction with the clay mineral is reversible. Finally, at 35 bar an uptake of 6.5 wt. % (1.59 mmol/g) is observed for Ni-Hec, at 33 bar an uptake of 3.6 wt. % (0.85 mmol/g) is observed for Li-Hec, and for Na-Hec no uptake is observed. As previously demonstrated by the surface area measurements on Ni-Hec,^19^ these clays provide a limited surface area; therefore, adsorption should largely occur in the interlayers, as is confirmed by the swelling behavior from the XRD measurements.

**Figure 5:**
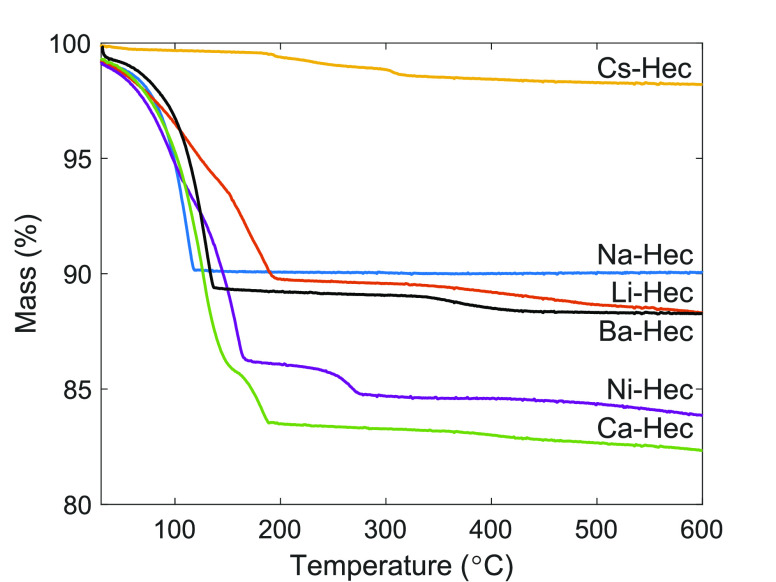
TGA of Li-Hec, Na-Hec, Cs-Hec, Ni-Hec, Ca-Hec, and Ba-Hec equilibrated at 43% RH.

Li-Hec does swell and adsorb in response to CO_2_. A possible explanation for this behavior is that Li-Hec may be a mixed-layer system where parts of the sample remain hydrated. Since Li^+^ is a small interlayer cation with a small ionic radius, one would expect it to obtain a basal spacing similar to that of Na-Hec when it is dehydrated (9.6 as opposed to 10.2 Å). In this case, as much as 20% of the interlayer space may still contain water, which is supported by TGA (Figure 5). Li-Hec shows a much more sluggish dehydration behavior compared to Na-Hec. For Na-Hec, only a very limited residual water population has been observed by INS (inelastic neutron spectroscopy) to be left in the sample after dehydration to 145 °C.^30^ Recent experiments on a Li-Hec from Corning^51^ found that dried Li-Hec contains tightly bound water molecules that have a significantly higher adsorption energy, 225 kJ/mol compared to 31 kJ/mol for the more loosely bound water population in the sample. This is also highlighted in another work on Corning Li-Hec,^52^ where water was observed by FTIR to desorb even above 200 °C. This water may then open up some of the layers sufficiently to allow for CO_2_ adsorption to happen. This explains the linear adsorption isotherm, where the sites available for adsorption increase proportionally with the concentration of CO_2_. The consequence of the open clay structure after the incorporation of CO_2_ would be a higher diffusion rate for water or for Li^+^ cations in the interlayers, eventually creating more sites for further CO_2_ adsorption. Increased diffusion of interlayer cations with increased basal spacing has been observed by NMR spectroscopy in other clay systems.^11^ The expansion of the interlayer space would be limited by the amount of water present in the system. This is supported by the observation of no clear single product phase in the PXRD measurements. As dehydration is hard to control for these systems, the sample in Figure 1 may be slightly more hydrated than the sample presented in Figure 3, as indicated by the clearer product phase. The present model where residual interlayer water assists the adsorption of CO_2_ is in line with previous reports and interpretations of clay swelling in response to CO_2_ with a hydration of between 0 and 1 WL.^12,23,27^

**Figure 6:**
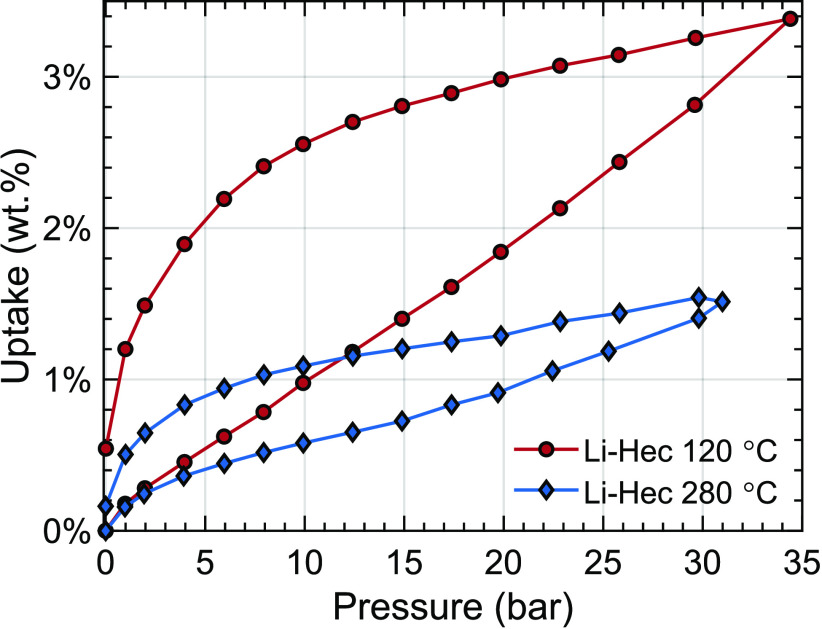
Gravimetric adsorption measurements of Li-Hec heated to 120 and 280 °C equilibrated at each pressure step for 1 h.

By additional heating of the Li-Hec sample to 280 °C, the gravimetric uptake is reduced significantly (Figure 6). At these temperatures, the sample should be more dehydrated as indicated by the TGA (Figure 5). Still, there are residual adsorption sites, even after heating at high temperatures. At these temperatures, the Hofmann–Klemen effect will occur,^37^ and some of the Li cations will migrate from the interlayer into the clay crystal structure.

For the remaining cations, the TGA shows sufficient dehydration behavior for Na-Hec and Ba-Hec at 150 °C (Figure 5), with the mass loss due to water prior to this temperature. For Cs-Hec, a low mass loss is observed due to its limited hydration because of the low hydration enthalphy.^53^ For Ca-Hec, a second mass loss is observed at 180 °C, and similar to Li-Hec, sluggish dehydration behavior is found. The XRD data also suggest that the system had some water left. However, Ca-Hec does not swell in response to CO_2_ under these experimental conditions. Repeated experiments on Ca-Hec have shown reproducible behavior in the response to CO_2_. The absence of swelling for Ca-Hec can possibly be explained by the difference in solvation energies for CO_2_ and H_2_O by the two cations Li^+^ and Ca^2+^. As demonstrated by Criscenti and Cygan,^54^ cations with a high solvation energy for water will partition preferentially into the H_2_O phase. For cations with a solvation energy more similar to those of H_2_O and CO_2_, the cations may partition into the CO_2_ fluid phase. Li^+^ has a more comparable solvation energy in CO_2_ or H_2_O and therefore may be prone to allowing CO_2_ to solvate the cation, whereas Ca^2+^ will prefer to interact with water molecules. Similar observations have been made on Mg-exchanged montmorillonite,^55^ where it has been observed that predrying Mg-montmorillonite samples at different temperatures significantly affected the uptake of CO_2_ and CH_4_. In line with our conclusions, this was argued to be due to tightly bound residual water remaining in the interlayers of the smectite, thus opening them up for CO_2_ adsorption.

The current observations confirm previous results for Na-Hec and other Na-smectites that the clay does not swell in response to CO_2_ without the presence of water.^11,29,30^ We can only speculate about the differences between the present work and refs (3), (4), and (6), but they may be attributed to the sample origin, charge, and phase purity as well as preparation methods including cation exchange (Li^+^ to Na^+^ for the commercial samples), drying procedures, and sample cells. Other studies have demonstrated that Cs^+^-exchanged smectite can incorporate CO_2_ when it is dehydrated under low vacuum at 50 °C^11^ and at 450 °C under vacuum,^20^ contrary to the present observations. At 50 °C, there may be some residual water left in the system; however, at 450 °C all water should be expelled. In ref (11), Wyoming MMT (SWy-2) is measured under supercritical conditions, and ref (20) demonstrates that the results still hold for the same clay mineral in the gaseous phase. We note that even at conditions corresponding to optimal uptake in ref (20) we do not observe swelling for Cs-Hec and Ba-Hec.

In this work, we have studied a high-quality synthetic clay mineral with respect to defects and impurities and with superior charge homogeneity.^34,56^ This is a very important issue to consider when comparing the present work to other publications. In addition, we can consider a number of other possible explanations for the discrepancies. First, the smectites in the previously mentioned works^3,4,11,29^ could have been insufficiently dehydrated. Second, clay–cation interactions, which have been suggested to be the dominant factor for CO_2_ adsorption,^11^ might be significantly different for our fluorinated Hec than for other smectites. For the works where MMT or other smectites have been studied, the clay–cation interaction is significantly different due to composition, charge density, charge homogeneity, and charge location within the layers. Such differences have been observed when examining SWy-2 and San Bernandino hectorite (SHCa-1), where correlations with the influence of cation polarizability on the CO_2_ adsorption were different between the two.^57^ Comparing our results on fully fluorinated Hec with those of other natural Hec’s, where there is a compositional difference due to a varying degree of F^–^ and OH^–^ substitutions, we would expect a higher CO_2_ adsorption in the present case from simulations,^10^ which is not the case. Third, for natural clays, side phases may significantly influence the uptake, for example, for Ni-Hec, where nickel hydroxide interstratified in the interlayer plays a dominant role in CO_2_ adsorption.^5^ In synthetic clay with high charge homogeneity, we have better control of the intercalation process and the history of the samples compared to natural systems. Finally, the layer charge for the current Hec is ∼40–80% higher than for the natural samples present in the other previously mentioned works,^11,20,29^ which result in a higher cohesion energy holding the platelets together and thus hindering swelling/uptake in response to CO_2_. Layer charge has been demonstrated for synthetic Ni-Hec to play a significant role in the adsorbed amount and pressure threshold for the swelling/adsorbing of CO_2_.^19^ In that case, a lower layer charge results in a lower pressure threshold, and it could be that the layer charge in this study is too high for the CO_2_ to be able to penetrate the layers. The present clay with Cs^+^ and Ba^2+^ has a higher charge and superior charge homogeneity compared to those of natural clays, which may explain why our synthetic case does not show CO_2_ adsorption for these ions. This is an aspect that warrants further study.

### DFT Calculations

**Figure 7:**
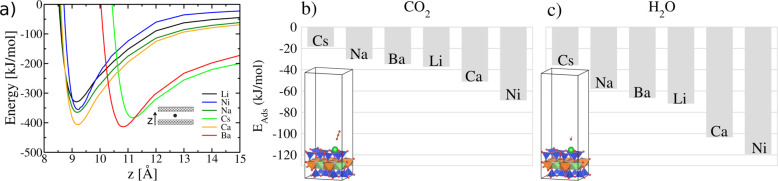
(a) Cohesion energy curves considering the different interlayer species. Adsorption energies of carbon dioxide (b) and water (c) molecules adsorbed above each interlayer atom in the clay material. The insets in panels (b) and (c) highlight the most stable configuration for the CO_2_ and H_2_O adsorption above the clay slab.

We systematically evaluated the cohesion energy and the layer separation distance by considering each of the six investigated species in the clay interlayer space (Figure 7a). The cohesion energy gives information about how difficult it is to separate the clay layers. With Li as the interlayer cation, we observed that the separation process is expected to be more easily performed compared to the other ones. Otherwise, clays with Ca or Ba interlayer atoms are the ones that may display the most substantial glue effect. Interestingly, the layer separation distance of the dried phase is affected by the species with larger atomic radii, i.e., Ba and Cs. The minor differences in the atomic radii of Ca, Na, Li, and Ni species do not influence this property. We also determined the adsorption energy for both carbon dioxide (Figure 7b) and water (Figure 7c) molecules interacting with the most favorable site in the clay slab surrounded by a vacuum. Among the adsorption sites, both molecules display the lowest adsorption energy by interacting with the interlayer cation. We observed that these molecules have the highest affinity for the clays that have Ni, Ca, and Li as the interlayer ions. Additional evidence may explain the swelling in clays that have Li and Ni atoms within their composition. Although the interaction between H_2_O, CO_2_, and clays with Ca in the interlayer space is favorable, one should consider that this atom also contributes to the high cohesion energy, making it more difficult for the clay to swell. Also, the differences observed for water and carbon dioxide adsorption energies reveal the higher affinity of H_2_O for the interlayer atom, to the detriment of CO_2_. Indeed, the swelling phenomena occurring in Hec may involve a competition between the cohesive energy and the differences observed between the adsorption energies of the molecules in the interlayer space, where the interlayer species drives both effects.

## Conclusions

We demonstrated that the selectivity for the intercalation of CO_2_ into fluorohectorite clay depends on the interlayer cation. Dehydrated fluorohectorite with interlayer cations Na^+^, Cs^+^, Ca^2+^, and Ba^2+^ did not swell or adsorb CO_2_ when dried sufficiently. For Li-fluorohectorite, on the other hand, we observed swelling and adsorption, which we relate to tightly bound residual water that we were unable to remove without reaching the temperature regime where the Hoffman–Klemen effect would significantly alter the clay structure. The experimental results are supported by DFT calculations.

A full understanding of the details of the interactions between CO_2_, residual water, and the interlayer cations, which may be obtained by spectroscopic measurements such as INS, IR, or Raman spectroscopy, would be helpful. In particular, this is needed to explain the interesting observations presented here for Li-Hec. To evaluate whether the discrepancies with the existing literature for the larger cations in this study are related to a cohesion energy that is too large due to the layer charge, further studies with Hec prepared at different layer charges will be conducted.
